# Chemical profile, antioxidant and antimicrobial activity of *Pinus heldreichii* Christ. Distributed in Bulgaria

**DOI:** 10.1016/j.heliyon.2023.e22967

**Published:** 2023-12-10

**Authors:** Ivanka Semerdjieva, Charles L. Cantrell, Valtcho D. Zheljazkov, Tzenka Radoukova, Lyubka H. Koleva-Valkova, Tess Astatkie, Miroslava Kačániová, Daniela Borisova

**Affiliations:** a Department of Botany and Agrometeorology, Agricultural University, Mendeleev 12, 4000 Plovdiv, Bulgaria; b Department of Plant and Fungal Diversity and Resources, Institute of Biodiversity and Ecosystem Research, Bulgarian Academy of Sciences, 1113 Sofia, Bulgaria; c Natural Products Utilization Research Unit, United States Department of Agriculture–Agricultural Research Service (USDA-ARS), University, MS 38677, USA; d Department of Crop and Soil Science, Oregon State University, 3050 SW Campus Way, 109 Crop Science Building, Corvallis, OR 97331, USA; e Department of Botany and Biological Education, University of Plovdiv Paisii Hilendarski, 24 Tzar Asen, 4000 Plovdiv, Bulgaria; f Department of Plant Physiology, Biochemistry and Genetics, Agricultural University, Mendeleev 12, 4000 Plovdiv, Bulgaria; g Faculty of Agriculture, Dalhousie University, Truro, NS B2N 5E3, Canada; h Institute of Horticulture, Faculty of Horticulture and Landscape Engineering, Slovak University of Agriculture, 949 76 Nitra, Slovakia; i School of Medical & Health Sciences, University of Economics and Human Sciences in Warsaw, Okopowa 59, 01 043 Warszawa, Poland; j Administration of Vrachanski Balkan Nature Park, Executive Forest Agency, Ministry of Agriculture and Food, 3000 Vratsa, Bulgaria

**Keywords:** *Pinus*, Tertiary relict, Balkan subendemic, Essential oils, Bulgaria, Biological activity

## Abstract

*Pinus heldreichii* Christ. (Bosnian pine), a Tertiary relict and Balkan sub-endemic, has not been comprehensively studied for its essential oil (EO) profile and bioactivity of its different plant parts. This study aimed to determine the EO yield, composition and antimicrobial activity from different parts of *P. heldreichii* at three different populations (mountains) in Bulgaria. Furthermore, the study assessed the antioxidant activities of plant tissue, including leaves (needles), twigs wood, male and female cones. The EOs yield from different plant parts ranged from 0.09 % (leaves) to 0.74 % (wood of twigs), with monoterpenes being the predominant class. Limonene, *α*-pinene, *β-*caryophyllene, germacrene D, *β-*pinene, and *β*-myrcene were detected in the EO extracted from all analyzed trees. However, these compounds were not found in the EO extracted from all plant parts of the same trees. Four chemical groups (chemotypes) were identified for EO from twigs, and three chemotypes were identified for EO from leaves. The chemotypes were based on the percent ratio of the main EO constituents (>5 %). Leaves tissue showed the highest values in terms of polyphenols and flavonoids, as well as higher ABTS radical scavenging activity, while the highest antimicrobial activity against *Staphylococcus aureus* subsp. *aureus* was seen in the EOs obtained from twigs. This is the first study to identify several chemotypes based on leaf and twigs EO of *P. heldreichii* distributed in Bulgarian flora. Furthermore, the EO of twigs tips (TT), male cones (MC), and wood of one-two-year-old twigs (WT) of the same trees were reported for the first time. The total polyphenol, flavonoid content, and radical scavenging activity of tissues of annual twigs wood and biennial twigs wood, leaf tissue, MC tissue, and the twigs tips tissue is also reported for the first time in the accessible literature. These findings highlight the potential of *P. heldreichii* to provide EOs with varying compositions and bioactivities, making them suitable for nutraceutical, pharmacological, and potentially food additive applications. Furthermore, the identification of chemotype accessions in this study suggests their selection for the development of new forest crop as a source for natural products with desirable composition and bioactivity.

## Graphical abstract

**Figure:**
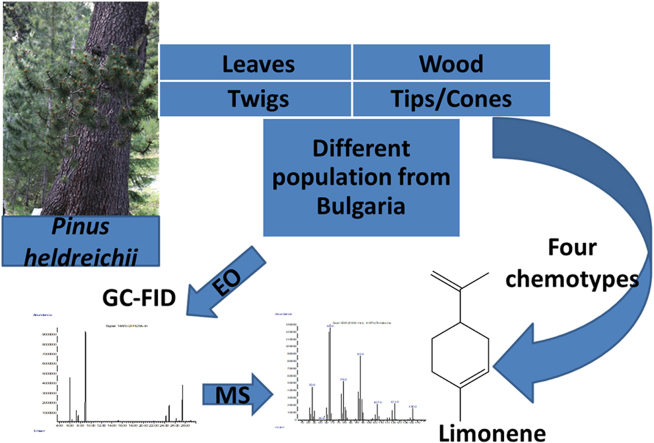

## Abreviations

MC: Male, cones (microstrobiles)
FC: Female, cones
WT: wood of one-two year-old twigs
T: whole twigs (leaves, wood, and twigs tip)
TT: twigs tips; L – leaves (needles) grinded
NL: not grinded leaves

## 1 Introduction

Pinaceae Lindl. species are a rich source of secondary metabolites, including monoterpenes, sesquiterpenes, diterpenes, phenolic compounds, and flavonoids [[1], [2], [3]]. Some *Pinus* species' essential oils (EO) and products have been used in folk medicines and phytotherapy due to their various therapeutic activities [[4], [5], [6]]. Exploring the phytochemical composition of plants has always been an area of interest for researchers and pharmacologists, particularly regarding the diversity of secondary metabolites in plants. The phytochemical composition of endemic plants with limited distribution provides a potential source for discovering new molecules and understanding the interaction between the plant and its environment [7].

*Pinus hеldreichii* (Bosnian pine), a survivor plant from the Tertiary era and a Balkan sub-endemic, is now mainly restricted to high altitude areas in the mountains of the Balkan Peninsula and is listed as the least concern species in 2016 by the IUCN Red List of Threatened Species™ [8]. However, its native populations are fragmented and exist under unfavorable conditions in Bulgaria, Albania, Bosnia, Greece, North Macedonia, Southern Italy and Serbia [9,10]. Given the limited distribution of *P. heldreichii*, research on its phytochemical composition of EO is mostly from Serbia, Greece, Bulgaria, Bosnia and Herzegovina, and Italy (Table 1). Most of the studies have been conducted on the territory of Serbia [[11], [12], [13], [14], [15], [16]]. The EO compositions were found to have significant diversity in terms of quantity and quality, with limonene and germacrene D being the most frequently reported major constituents of *P. heldreichii* EO (Table 1). However, the compositions of EO were found to vary depending on the extraction method used, the time of sample collection and duration of distillation. As a result of the review of the literature report, several knowledge gaps were identified: (1) Some of the authors cited in Table 1 examined many trees (>30), but combined the results and presented the result as average values; these did not appear to represent the range of EO in natural populations; (2) Some authors studied and reported only one individual per population, one single sample. Obviously, one single sample per population could not be regarded as representative; (3) Most of the authors cited in Table 1 examined only one part of the tree (twigs; or leaves; or F cones); (4) In the accessible literature, there was no data on EO composition of twig tips (TT), the wood of one-two-year-old twigs (WT), and male cones (MC); (5) We did not find comprehensive study on the plant parts EO from one individual tree; (6) Much of the published data makes no mention of repetitions and lacks proper or any statistics. The indicated gaps in the research on the species are a prerequisite for misinterpretation of the results. Therefore, there is a need for a comprehensive study on the EO profile and bioactivity of different parts of the same tree of *P. heldreichii*, such as L, T, WT, TT, MC, and FC. The *Pinus* genus is widely recognized for its abundance of secondary metabolites that offer valuable properties. Most research focuses on the antioxidant potential of EO obtained from various conifers [[17], [18], [19]]. They pay attention to the composition of the oils and their biological effects [20,21]. A very small number of studies focused on the properties of extracts with different solvents obtained from different parts of conifers [22], and even fewer related to *P*. *heldreichii* [9,23]. While numerous studies have focused on establishing the chemical composition and biological activity of essential oils from the leaves of various *Pinus* species [20,24], there is a dearth of information on crude extracts and their potential. Polyphenols are a class of secondary metabolites that play a crucial role as antioxidants, protect against herbivores and pathogens, repel insects, and attract pollinators. Flavonoids have a broad range of biological effects, including antioxidant, anti-inflammatory, vasorelaxant, antimicrobial, antiviral, anticarcinogenic, and antimutagenic properties [17,18]. Antiradical activity refers to the ability of compounds to react with free radicals, and test systems utilizing stable free radicals such as DPPH, ABTS, etc., can provide valuable information regarding the radical scavenging or antiradical activity of plant parts or tissues. Plants with high levels of antiradical compounds have the potential to serve as valuable sources of medicinal ingredients.

**Table 1:** Literature data of research of *Pinus heldreichii* EO.

Reference	Country	Studied part	Collection Time	DT or E^a^	EO yield	The compounds in %
**Maric et al**. [9]	Herzegovina, Bosnia	Needles	August	HD	4.5 mg g^−1^	limonene (52.8); germacrene D (15.8); *α*-pinene (10.2); *trans*-caryophyllene (7.7); *β*‐ pinene (3.0);
**Bojović et al**. [11]	Serbia	Needles	August 15–20	n-pentane	no	limonene (9.01–24.13); germacrene D (21.18–33.46); *α*-pinene (8.48–11.8); *β*‐caryophyllene (9.73–15.10); Δ3-careen (0.12–8.49); *β*‐pinene (2.81–3.56);
**Menković et al**. [12]	Serbia	Young shoots,	Summer	HD	1.25 %	*α*-pinene (10.76–15.42); limonene (41.05–63.46); *β*-myrcene (2.62–3.33); *β-*cubebene (0.15–7.25); *trans*-caryophyllene (0.61–2.81)
		Cones			0.85 %	*α*-pinene (10.39–12.78); limonene (75.90–77.75); *β*-myrcene (2.49–2.89); *β-*cubebene (2.51–2.62); *trans*-caryophyllene (2.18–2.38);
**Simić et al**. [13]	Serbia	Needles	Summer	HD	no	limonene (20.26); germacrene D (42.0–42.6); *β*‐caryophyllene (10.58–13.3);
**Nikolić et al**. [14]	Montenegro, Serbia	Needles	Late summer to early fall	pentane	no	limonene (26.3); germacrene D (13.5); *α*-pinene (17.5); *β*‐caryophyllene (10.5)
**Nikolić et al**. [15]	Scardo-Pindic Mountains Oslak and Galicica	Needles	Late summer	pentane	no	limonene (27.1); germacrene D (28.7); *α*-pinene (16.2); *β*‐caryophyllene (6.9); *β*‐pinene (5.2);
**Rajčević et al**. [16]	Scardo-Pindic mountain system, Montenegro	Twigs with Needles	Late summer	n-pentane	no	limonene (23.2–31.9); germacrene D (11.4–32.1); *α*-pinene (11.1–22.2); *β*‐caryophyllene (4.5–11.4); *β*‐pinene (3.6–7.2);
**Graikou et al**. [20]	North Greece	Wood	Not shown	HD	no	limonene (28.7); *α*-pinene (6.43); cembrene (23.82);
**Basholli-Salihu et al**. [21]	Kosovo	Needles	July to September	HD	0.2–0.3 %	limonene (43.9); germacrene D (17.17); α-pinene (10.57); *β*‐caryophyllene (4.4); caryophyllene-oxyde (3.11);
**Petrakis et al**. [25]	Katara, Central Greece	Needles	Not shown	HD	no	limonene (34.3); germacrene D (12.8); *α*-pinene (13.8); *β*‐caryophyllene (8.4); camphene (1.5); Δ3-carene (2.8); *β*‐pinene (4.2); myrcene (2.5); aristolene (6.0);
**Naydenov et al**. [26]	Bulgaria	Needles	Winter	diethyl and petrol ether	no	*α*-pinene (16.92–18.60 %); camphene (1.86–2.23 %); *β*-pinene (5.07–6.49 %); *δ*-3-carene (3.20–4.96 %); limonene (36.90–48.20 %); *β*-farnesene (4.73–7.64 %); *γ*-muurolene (14.85–22.87 %);
**Bonesi et al**. [27]	Calabria, Italy	Needles	Full flowering stage	HD	0.2 %	limonene (7.8); germacrene D (0.7); *α*-pinene (24.2); *β*‐pinene (8.4)
**Ioannou et al**. [28]	Botanical garden, Greece	Needles	Not shown	HD	no	limonene (23.7 %), germacrene D (21.3 %); *β*-3-carene (18.6 %); *α*-pinene (11.1 %); *β*-caryophyllene (8.6 %);
**Mitić et al**. [29]	Bulgaria, Pirin	Needles	September	HD	0.28 %	limonene (34.4); germacrene D (17.3); α-pinene (23.8); *β*‐caryophyllene (11.1); *β*‐pinene (8.8);
**Semerdjieva et al**. [30]	Bulgaria	Twigs with Needles	July	HD	0.4 %	limonene (76.9 %–77.0 %); *α*-pinene (12.47 %–15.54 %);

a E − extraction; DT – distillation type; HD – hydrodistillation; ST – steam distillation; OS – organic solvent.

These knowledge omissions have led us to establish specific research objectives. The objective of this study was to determine the variability of the EO composition, antimicrobial and antioxidant activities, and phenolic and flavonoid content of different parts tissue of *P. heldreichii* in Bulgarian populations. The working hypothesis was that the EO from different plant parts of the same tree would have similar compositions. Furthermore, this study identified the phenolic and flavonoid content of different tissue parts' of species, and assessed antimicrobial activity of the EOs.

## 2 Materials and methods

### 2.1 Collection of the plant material

**Fig. 1:**
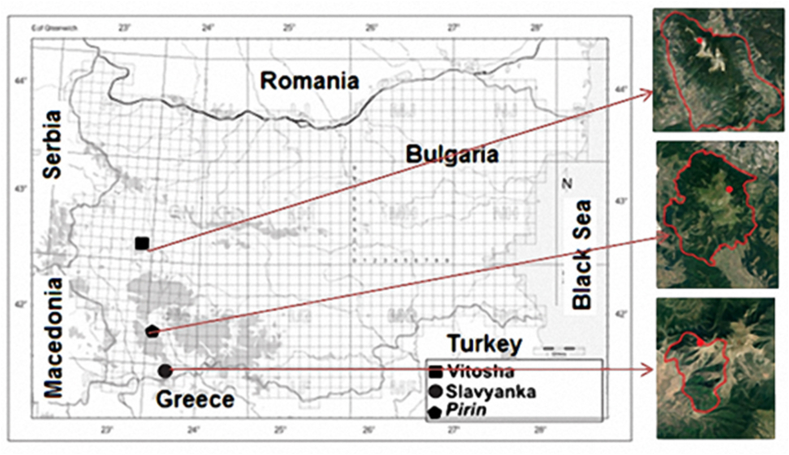
Map of the distribution and collected samples of *Pinus heldreichii* in Bulgaria.

Samples of *Pinus heldreichii* were collected in July 2–5, 2020 from natural populations in Pirin Mountain, National Park and Slavyanka Mountain, as well as from Vitosha Mountain Park, where the species is introduced (Fig. 1). All samples were collected under an official permit (# 185 [1]/March 11, 2020) from the directorates of the respective parks. The samples were taken from one tree in Vitosha Park (T1) three trees per population in Slavyanka Mountain (T2-T4), and Pirin Mountain (T5-T7). The environmental characteristic in Table 2 of the three populations were described according to Geograpgy of Bulgaria [31]. The samples, which consisted of twigs with leaves, were deposited at the herbarium SOA at Agricultural University, Plovdiv, Bulgaria for future reference [32].

**Table 2:** Details of localities of samples of *Pinus heldreichii* in Bulgaria.

Population, tree (Т) number	Coordinates/Exposure	Environmental characteristic	masl	Plant part	Used samples/g
Vitosha Park, T1	42.59264°N 23.30108°E	Аndesites rock; *Cambisols* soils; Continental climate; south/east exposure	1807	MC	60
				WT	70
				T	100
				L	100
Slavyanka Mountain, T2	41.40884°N 23.61114°E	Continental-Mediterranean climate area; Marble rock; *Rendzinas* soils; south/east; northeast	1556	T	100
				L	100
				MC	75
				TT	100
Slavyanka Mountain, T3	41.40890°N 23.61127°E		1556	MC	75
				T	100
				L/NL	90
				TT	100
				WT	100
Slavyanka Mountain, T4	41.40000°N 23.60950°E		1570	T	100
				L/NL	100
				WT	100
				MC	50
Pirin Mountain, T5	41.76586°N 23.42228°E	Marble limestone (calcareous) rocks; *Rendzinas* soils; Northeast exposure/south/east; The Continental-Mediterranean climate area;	1833	MC	70
				FC	80
				TT	45
				T	100
				WT	70
				L	100
Pirin Mountain, T6	41.76563°N 23.42054°E		1861	T	100
				WT	55
				MC	75
				L	100
				TT	30
Pirin Mountain, T7	41.76464°N 23.41928°E		1929	T	100
				L	100
				TT	55
				WT	55

MC - Male, cones (microstrobiles); FC – Female, cones (unripe); WT - wood of one-two year-old twigs; TT – twigs tips; T - whole twigs (leaves, wood, and twigs tip); L – leaves (needles) grinded; NL – non grinded leaves (needles).

### 2.2 Essential oil (EO) extraction

To extract the essential oil from *P. heldreichii*, the collected materials, such as one-two-year old twigs were first dried in a well-aerated environment in the laboratory of Botany and Agrometeorology at Agricultural University, Plovdiv. After that, each sample per tree was divided into subsamples as follows: leaves (needles) (L) grinded, wood of one-two-year old twigs (twigs - without leaves and tips) (WT), the twigs tips (TT), and whole twigs (leaves, wood, and twigs tips) (T). Where male (M, microstrobiles) and female (F) unripe cones (MC; FC) were present in the samples, they were also analyzed. The EO of air-dried L, MC, FC, WT, TT, T of *P. heldreichii* were extracted by Clevenger type hydrodistillation for 3 h. Two-liter (L) distillation units (https://en.laborbio.com/) were used. Before each distillation, the samples were cut into small pieces (0.5–1 cm), then grinded in a blender “Fagor” for 2–3 min. All samples were grinded mixed with 0.800 L of water, and then 0.800 L water was added to the 2-L distillation units. Generally, we blended in water to avoid and eliminate EO loss and it is based on our previous experience [30,33]. In total, 1.600 L water was used for each individual distillation. All samples were extracted in two replicates. To understand and demonstrate differences in EO yield and composition due to grinding, we included the two leaves samples (T3, T4) without grinding, only cut.

The EO was transferred into 4-mL vials, which were then placed in a freezer. Subsequently, it was separated from the water. The analytical scale was used to measure the EO, which was subsequently stored at 4–6 °C until the analysis. The oil content (yield) was reported based on weight (g) to dry mass.

### 2.3 Gas chromatography (GC), mass spectrometry (MS) analyses - flame ionization detection (FID)

The isolated EO from all *P. heldreichii* samples were analyzed via gas chromatography with simultaneous mass spectrometry and flame ionization detection (GC-MS-FID) by dissolving 50 μL of EO into a 10 mL volumetric flask and brought to volume in CHCl_3_. GC-MS-FID conditions were as previously reported [30]. Post-column splitting was performed (50 % FID/50 % MS) and all compounds were identified by Kovat and/or Retention Index analysis [34], direct comparison of MS data and analyte retention time to that of authentic standards and comparison of mass spectra with those reported in the NIST mass spectral database. Commercial standards of *α*-pinene, camphene, *β*-pinene, *β*-myrcene, *α*-phellandrene, limonene, bornyl acetate, and *β*-caryophyllene were purchased from Sigma-Aldrich (St. Louis, MO, USA). Germacrene D was confirmed using Adams library and Kovat and/or Retention Index analysis [34]. Compounds quantified by performing area percentage calculations based on the total combined FID area.

### 2.4 Total polyphenol, flavonoid content and radical scavenging activity of different parts of *Pinus heldreichii* (annual twigs wood, biennial twigs wood, leaf, M cones, and twigs tips)

#### 2.4.1 Plant material extraction

One gram of dried leaves (needles), annual twigs wood, biennial twigs wood, M cones, and twigs tips of samples collected from Slavyanka Mountain were chopped to small 2 mm pieces and mixed with 10 ml 70 % acidic methanol in tube with cap. Four replications were made. The tubes were placed in an ultrasonic bath for 30 min, and then were kept at room temperature in the dark for 24 h extraction. After the extraction, the samples were centrifuged at 6708 g for 10 min, and the supernatant was used for analysis.

#### 2.4.2 Total phenol content analysis of annual WT, biennial WT, leaf, MC, and TT

Quantitative determination of total phenols was performed with Folin-Ciocalteu reagent by the method of Singleton and Rossi [35], with minor modifications [36]. The absorbance was detected at 760 nm with UV/Vis spectrophotometer Spectroquant Pharo300 (Germany). The results were calculated from a standard curve and expressed as mg of gallic acid equivalents per gram dry weight.

#### 2.4.3 Total flavonoid content analysis of annual twigs wood, biennial twigs wood, leaf, M cones, and twigs tips

Quantitative flavonoid contents were determined with aluminum trichloride [37]. The absorbance was detected spectrophotometrically at the wavelength of 510 nm. The total amount of flavonoids was calculated from a standard quercetin curve and was expressed as mg of quercetin equivalents per gram dry mass.

#### 2.4.4 Antiradical activity of annual twigs wood, biennial twigs wood, leaf, M, cones, and twigs tips

##### 2.4.4.1 DPPH• assay

To prepare the 2,2-diphenyl-*l*-picrylhydrazyl (DPPH) reagent, 0.012 g was dissolved in 100 mL of absolute ethanol and placed in an ultrasonic bath two times for 15 min. This solution was used to determine the antiradical activity of plant extracts by the method of Brand-Williams [38] with minor changes. For this purpose to 50 μL of sample, 2.95 mL of DPPH• solution was added and the absorption was recorded after 5 min at 517 nm against absolute alcohol. A parallel blank sample containing acidic methanol instead of an extract was tested. The ability to scavenge DPPH radical was expressed as mg Trolox equivalent antioxidant capacity (TEAC) calculated by standard curve, or expressed as % decolorization calculated using the formula:

DPPH inhibition (%) = (100-(Abs_5min_/Abs_blank_)*100).

##### 2.4.4.2 ABTS• + assay

The ABTS***• +*** assay included the preparation of stock and working solutions and following an approved procedure. The method was a modification of Xiao et al. [39] method. Stock solutions of ABTS• (7 mM) and K_2_S_2_O_8_ (140 mM) were prepared and kept in dark (0–4 °C). Five milliliters of ABTS (7 mM) and 88 μL of K_2_S_2_O_8_ (140 mM) stock solutions were mixed and kept at room temperature for 16 h in the dark to make an ABTS***• +*** reaction solution. After activation, the ABTS***• +*** reaction solution was diluted with distilled water to give the absorbance 0.700 ± 0.05 at 734 nm.

##### 2.4.4.3 ABTS• +assay procedure

A 100 μL sample and 2.900 mL ABTS***• +*** reaction solution were mixed in tubes. The tubes were incubated for 5 min, and then the absorbance was measured at 734 nm wavelength. Distilled water was used as the blank control. The ABTS***• +*** radical scavenging activity was expressed as mg Trolox equivalent antioxidant capacity (TEAC) calculated by standard curve or as % decolorization calculated as:

ABTS***• +*** inhibition (%) = (100-(Abs_5min_/Abs_blank_)*100)

All assays were carried out in four replicates.

### 2.5 Antimicrobial activity of essential oils (EO) of *Pinus heldreichii*

#### 2.5.1 Microorganisms tested

Three Gram-positive bacteria *(Staphylococcus aureus* subsp*. aureus* CCM 2461*, Listeria monocytogenes* CCM 4699*, Bacillus cereus* CCM 2010*),* three Gram-negative bacteria *(Salmonella enterica* subsp*. enterica CCM 3807, Pseudomonas aeruginosa* CCM 1959*, Escherichia coli* CCM 3988*),* and three yeasts *(Candida albicans* CCM 8186*, Candida glabrata* CCM 8270*, Candida tropicalis* CCM 8223*),* were used for the antimicrobial activity testing in our study from the Czech collection of microorganisms (CCM, Brno, Czech Republic).

#### 2.5.2 Disc diffusion method

For the agar disc diffusion method, 100 μL of bacterial suspensions following incubation on Mueller-Hinton agar (MHA, Oxoid, Basingstoke, UK) and yeast suspensions on Sabouraud Dextrose agar (SDA, Oxoid, Basingstoke, UK) were dispersed. The value of 0.5 McFarland suggests that there are 1.5 × 10^8^ adhering colonies per milliliter of forming units (CFU). The filter paper discs (6 mm in diameter) were placed on the inoculated MHA or SDA, respectively, with 15 μL of the EO infused on them. The MHA and SDA were held at 4 °C for 2 h before being kept at 37 °C and 25 °C, respectively, for 24 h in an aerobic environment. Positive controls included the antibiotics Cefoxitin and Gentamicin (30 g/disc, Oxoid, Basingstoke, UK), as well as the antifungal Fluconazole (30 g/disc, Oxoid, Basingstoke, UK). For each testing, three replications were chosen.

### 2.6 Statistical analyses

#### 2.6.1 Statistical analyses of essential oils

The effects of (1) Population (3 levels: Pirin, Slavyanka, and Vitosha), and (2) Plantpart nested in population (34 levels: WT, T1-T7; FC, T1; L, T1-T7; MC, T1-T6; TT, T1-T7; T, T1-T7; NL, T3, T4) on oil yield, *α*-pinene, *β*-pinene, *β*-myrcene, limonene, *β*-caryophyllene, germacrene D, monoterpenes (MT %), and sesquiterpenes (ST %) was determined using a Nested design where plant part is nested in population. The components of the model are shown in the Source of Variation row of Table 3. The multiple means comparison results are shown in Table 4, Table 5.

**Table 3:** ANOVA *p*-values that show the significance of the effects of Population and Plantpart(Popultation) (Plantpart nested in Population) on 9 response variables. Significant effects that require multiple means comparison are shown in bold. MT% = monoterpenes (in %); ST% = sesquiterpenes (in %).

Source of variation	EO yield	*α*-Pinene	*β*-Pinene	*β*-Myrcene	Limonene	*β*-Caryophyllene	Germacrene D	MT%	ST%
Population	0.001	0.001	0.001	0.014	0.001	0.001	0.005	0.003	0.003
Plantpart (Population)	**0.001**	**0.001**	**0.001**	**0.001**	**0.001**	**0.001**	**0.001**	**0.001**	**0.001**

**Table 4:** Mean EO yield (%), and concentration (%) of MT, and ST, and *α*-pinene and *β*-pinene, obtained from the 34 combinations of plant part and population.

Population	**Plant part**	**Tree № (T)**	**EO yield**	**MT%**	**ST%**	***α*-Pinene**	***β*-Pinene**
Vitosha Park	WT	T1	0.74 a	90.8 abc	5.29 hij	8.94 c-f	0.60 klm
Slavyanka Mountain	WT	T2	0.14 f	51.8 d-h	13.41 f-j	3.01 fg	0.34 lm
Slavyanka Mountain	WT	T3	0.14 f	51.8 d-h	13.41 f-j	3.01 fg	0.34 lm
Slavyanka Mountain	WT	T4	0.11 f	29.7 gh	2.25 ij	0.59 g	0.00 m
Pirin Mountain	WT	T5	0.72 a	91.8 ab	5.25 hij	11.19 c-f	3.48 d-h
Pirin Mountan	WT	T6	0.23 c-f	80.3 a-d	4.40 hij	5.94 d-g	2.96 f-k
Pirin Mountain	WT	T7	0.34 b-f	69.8 a-e	11.46 f-j	5.99 d-g	0.91 i-m
Pirin Mountain	FC	T5	0.33 b-f	64.5 a-g	11.95 f-j	6.97 c-g	0.58 klm
Vitosha Park	L	T1	0.2d ef	31.6 fgh	61.05 a	7.97 c-f	2.92 f-k
Slavyanka Mountain	L	T2	0.39 b-f	56.9 b-h	33.83 b-f	44.61 a	8.98 a
Slavyanka Mountain	L	T3	0.25 c-f	50.3 d-h	45.94 abc	14.68 b-e	5.88 bcd
Slavyanka Mountain	L	T4	0.15 ef	75.9 a-d	20.57 c-j	19.72 bcd	6.27 BCE
Pirin Mountain	L	T5	0.15 ef	55.7 b-h	29.56 c-h	8.71 c-f	3.88 c-g
Pirin Mountain	L	T6	0.09 f	56.9 b-h	40.83 a-e	6.02 d-g	0.00 m
Pirin Mountain	L	T7	0.09 f	39.6 e-h	42.26 a-d	10.91 c-f	3.33 e-i
Vitosha Park	MC	T1	0.48 a-e	90.6 abc	4.18 hij	17.51 bcd	0.89 i-m
Slavyanka Mountain	MC	T2	0.33 b-f	83.9 a-d	1.20 j	21.01 BCE	1.22 h-m
Slavyanka Mountain	MC	T3	0.15 f	89.6 abc	3.07 ij	9.94 c-f	0.79 j-m
Slavyanka Mountain	MC	T4	0.15 f	97.5 a	0.00 j	16.44 bcd	0.00 m
Pirin Mountain	MC	T5	0.12 f	80.0 a-d	10.21 f-j	10.42 c-f	3.13 f-j
Pirin Mountain	MC	T6	0.14 f	85.9 a-d	1.49 j	12.82 c-f	3.04 f-k
Slavyanka Mountain	TT	T2	0.22 def	71.9 a-e	12.89 f-j	32.22 ab	5.87 bcd
Slavyanka Mountain	TT	T3	0.16 ef	60.6 b-h	29.93 c-h	7.85 c-f	2.78 f-l
Pirin Mountain	TT	T5	0.20 def	79.2 a-d	6.74 g-j	9.34 c-f	2.92 f-k
Pirin Mountain	TT	T7	0.24 c-f	77.1 a-d	13.96 f-j	7.40 c-g	1.22 h-m
Vitosha Park	T	T1	0.60 ab	76.3 a-d	17.57 d-j	13.45 b-f	2.65 f-l
Slavyanka Mountain	T	T2	0.55 abc	60.0 b-h	27.94 c-i	46.63 a	7.26 ab
Slavyanka Mountain	T	T3	0.37 b-f	60.3 b-h	32.85 b-g	9.85 c-f	3.74 d-g
Slavyanka Mountain	T	T4	0.51a-d	69.5 a-e	21.05 c-j	9.82 c-f	2.44 f-m
Pirin Mountain	T	T5	0.26 c-f	88.5 abc	8.94 f-j	13.72 b-f	4.83 b-f
Pirin Mountain	T	T6	0.25 c-f	80.2 a-d	14.87 e-j	14.49 b-e	5.63 b-e
Pirin Mountain	T	T7	0.21 def	51.9 d-h	42.27 a-d	8.30 c-f	2.39 f-m
Slavyanka Mountain	NL	T3	0.12 f	24.8 h	56.41 ab	4.15 efg	2.51 f-l
Slavyanka Mountain	NL	T4	0.10 f	54.9 c-h	29.56 c-h	8.71 c-f	2.14 g-m

Within each column, means sharing the same letter are not significantly different. Four or more letters (e.g., abcd) are shown as a-d. WT - wood of one-two year-old twigs; FC – Female, cones (unripe); L – leaves (needles) grinded; MC - Male, cones (microstrobiles); TT – twigs tips; T - whole twigs (leaves, wood, and twigs tip); NL - non-grinded leaves. The letter groupings were done using Tukey's studentized range test method at 5 % level of significance.

Camphene, *β*-myrcene, and bornyl acetate were found in lower amounts, less than 5 % (Table S1). Overall, the results of the study indicate significant variability in the EO composition of *P. heldreichii* in different parts of the tree and in trees from different populations.

**Table 5:** Mean concentration (%) of *β*-myrcene, limonene, *β*-caryophyllene, and germacrene D obtained from the 34 combinations of plant part and population.

Population	**Plant part**	**Tree № (T)**	*β*-Myrcene	**Limonene**	*β*-Caryophyllene	Germacrene D
Vitosha Park	WT	T1	2.11 abc	79.1 ab	3.82 e-j	1.46 g-k
Slavyanka Mountain	WT	T2	0.92 b-e	48.6 d-g	6.46 c-h	0.09 ijk
Slavyanka Mountain	WT	T3	0.90 b-e	47.5 d-g	5.76 c-h	7.02 d-h
Slavyanka Mountain	WT	T4	0.12 de	28.2 g-m	1.91 g-j	0.06 ijk
Pirin Mountain	WT	T5	2.08 abc	22.4 j-o	4.30 d-j	0.93g-k1
Pirin Mountan	WT	T6	0.54 cde	60.4 a-f	1.48 hij	2.27 g-j
Pirin Mountain	WT	T7	1.98 abc	60.5 a-f	6.42 c-g	4.96 d-i
Pirin Mountain	FC	T5	1.58 a-e	53.7 c-f	4.73 d-i	6.99 d-h
Vitosha Park	L	T1	0.81 b-e	19.0 l-o	17.84 ab	43.11 a
Slavyanka Mountain	L	T2	1.01 b-e	1.9 no	4.38 d-i	29.41 a-d
Slavyanka Mountain	L	T3	1.21 a-e	23.5 h-n	11.85 a-d	39.07 abc
Slavyanka Mountain	L	T4	2.71 a	46.0 e-h	13.48 abc	7.06 c-h
Pirin Mountain	L	T5	0.00 e	42.7 f-k	21.99 ab	6.97 d-h
Pirin Mountain	L	T6	0.00 e	20.9 k-o	29.98 a	40.80 ab
Pirin Mountain	L	T7	1.39 a-e	24.0 h-n	10.85 a-e	31.33 a-d
Vitosha Park	MC	T1	1.75 a-d	69.8 a-d	2.88 f-j	1.29 g-k
Slavyanka Mountain	MC	T2	1.33 a-e	58.0 b-f	0.91 j	0.05 jk
Slavyanka Mountain	MC	T3	1.57 a-e	75.3 abc	1.98 g-j	1.07 g-k
Slavyanka Mountain	MC	T4	0.00 e	81.1 a	0.00 k	0.00 k
Pirin Mountain	MC	T5	1.29 a-e	39.7 f-l	5.83 c-h	3.71 e-j
Pirin Mountain	MC	T6	1.42 a-e	66.3 a-e	1.06 ij	0.08 ijk
Slavyanka Mountain	TT	T2	1.79 abc	27.3 g-m	3.13 f-j	9.75 a-g
Slavyanka Mountain	TT	T3	1.20 a-e	45.4 e-i	6.40 c-g	23.72 a-f
Pirin Mountain	TT	T5	1.31 a-e	23.2 i-n	6.22 c-g	0.35 h-k
Pirin Mountain	TT	T7	2.14 abc	55.6 c-f	6.05 c-g	7.90 b-h
Vitosha Park	T	T1	1.97 abc	57.6 b-f	6.53 c-g	10.40 a-g
Slavyanka Mountain	T	T2	1.04 b-e	3.8 no	4.59 d-i	22.91 a-f
Slavyanka Mountain	T	T3	1.56 a-e	45.0 e-j	7.43 b-f	25.39 a-e
Slavyanka Mountain	T	T4	2.25 ab	69.5 a-d	7.22 b-f	10.74 a-g
Pirin Mountain	T	T5	2.07 abc	22.0 k-o	5.79 c-h	3.08 f-j
Pirin Mountain	T	T6	1.69 a-d	58.1 b-f	4.26 d-j	10.04 a-g
Pirin Mountain	T	T7	1.44 a-e	39.7 f-l	11.77 a-d	28.99 a-d
Slavyanka Mountain	NL	T3	0.00 e	16.3 mno	17.01 ab	39.40 ab
Slavyanka Mountain	NL	T4	0.88 b-e	42.7 f-k	21.99 a	6.97 d-h

Within each column, means sharing the same letter are not significantly different. Four or more letters (e.g., abcd) are shown as a-d. WT - wood of one-two year-old twigs; FC – Female, cones (unripe); L – leaves (needles) grinded; MC - Male, cones (microstrobiles); TT – twigs tips; T - whole twigs (leaves, wood, and twigs tip); NL - non-grinded leaves. The letter groupings were done using Tukey's studentized range test method at 5 % level of significance.

The analysis was conducted using the GLM procedure of SAS 9.4 [40]. For each response variable, the validity of model assumptions on the error terms was verified by examining the residuals as described in Montgomery [41]. When normal distribution assumption is violated, an appropriate transformation was applied; however, their means reported in the tables are backtransformed to the original scale. The effects of Population and Plantpart nested in Population on camphene, *α*-phellandrene, and bornyl acetate were not determined because there were lots of zeros that would not allow to meet the normal distribution assumption on the error terms.

Since Plantpart nested in Population (Plantpart(Population)) effect was highly significant (*p* < 0.01) in all response variables, multiple means comparison was conducted using the Tukey's studentized range test method at 5 % level of significance to compare the 34 combinations of Population and Plantpart. This method was used to protect the inflation of Type I experimentwise error rate due to the large number of means being compared. Even if the effect of Population is significant on all response variables, if the effect of Plantpart(Population) is significant, multiple means comparison was done only on Plantpart(Population) because the differences among the plant parts vary with the Population.

#### 2.6.2 Statistical analysis of total polyphenol and flavonoid content and radical scavenging activity

The effect of Plant part (6 levels: annual wood, biennial wood, leaf, male cones, and twigs tips) on Phenols, Flavonoids, DPPH inhibition, DPPH TE, ABTS inhibition, and ABTS TE was determined using a one-way analysis of variance (ANOVA). The analysis was completed using the GLM Procedure of SAS 9.4 [40], and the validity of model assumptions was verified by examining the residuals as described in Montgomery [41]. Since the effect of Plant part was highly significant (*p*-value <0.01) on all six response variables, multiple means comparison was completed using Tukey's studentized range test method at the 5 % level of significance.

#### 2.6.3 Statistical analysis of antimicrobial activity of essential oils

Descriptive statistics (the mean and the standard deviation) of inhibition of halos diameters of *Staphylococcus aureus* subsp. *aureus* (SA); *Listeria monocytogenes* (LM); *Bacillus cereus* (BC); *Salmonella enterica* subsp*. enterica* (SE); *Pseudomonas aeruginosa* (PA); *Escherichia coli* (EC); *Candida albicans* (CA); *Candida glabrata* (CG); and *Candida tropicalis* (CT) are presented and discussed.

## 3 Results

### 3.1 EO yield of wood of one-two-year-old twigs (WT), leaves (L), the twigs tips (TT), cones (M, F)(MC, FC), and whole twigs (wood, leaves, twigs tips) of P. hеldreichii

The variability of EO yield of *P. heldreichii* was presented in Table 3, Table 4 The EO yield exhibited significant variability, ranging from 0.09 % for L to 0.74 % for WT. Notably, the highest EO yield (0.74 %) was obtained from the WT, whereas the leaves yielded significantly less. Based on the significant differences among EO yields, the different parts of *P. heldreichii* were ranked as follows: WT, T1 (Vitosha Park, 0.74 %) > WT, T5 (Pirin, 0.72 %) > T, T1 (Vitosha 0.6 %) > T, T2 (Slavyanka 0.55 %) > T, T4 Slavyanka 0.51) > MC, T1 (Vitosha 0.48) > TT and L. It is worth noting that no trends were observed between EO yields and the geographical location of populations. For instance, although the samples of WT from Slavyanka (T2) and Vitosha (T1) had high EO yields, the yields from other trees in the same population, such as Slavyanka (T3, T4), were approximately two times lower. Similarly, variations in yields were observed for others research parts of the species, including T, TT, and L samples. This suggests that the individual physiological and genetic characteristics of *P. heldreichii* may influence the EO yield because the trees in population were in the same condition, and the samples and retrieval of EO were in the same ways.

### 3.2 Essential oil composition of wood of one-two-year-old twigs (WT), leaves (L), non-grinded leaves (NL), the twigs tips (TT), cones (M, F)(MC, FC), and whole twigs (wood, leaves, twigs tips) of P. hеldreichii

The essential oil (EO) composition of various parts of *P. heldreichii* (including WT, T, L, MC, FC, TT, NL) was analyzed and the multiple means comparison results are presented in Table 4, Table 5. The predominant class of compounds in most samples was monoterpenes, with limonene, *β*-caryophyllene, *α*-pinene, and germacrene D being the most commonly found compounds (Table 4, Table 5). Overall, the main components of EO varied both between individual parts and between different trees.

The concentration of limonene varied depending on the tree and the part of the tree. The highest concentration of limonene was found in MC, T4 from Slavyanka, with a percentage of 81.1 %, while the lowest was found in L, T2 from Slavyanka, with a percentage of 1.9 %.

Similarly, the concentration of *β*-caryophyllene ranged from 0.0 % in MC, T4 from Slavyanka to 29.98 % in L, T6 from Pirin (Table 5). In most leaves' samples (other than L, T2) *β*-caryophyllene was higher than in the other studied tree parts.

Germacrene D was not detected in some samples, but was found in high concentrations in the leaves of *P. heldreichii*, ranging from 39.07 % to 43.11 % (Table 5).

*α*-Pinene was found in high concentrations on T2 collected from Slavyanka Mountain as follows: T (46.63 %) > L (44.61 %) > TT (32.22 %) > MC (21.01 %), while in WT, T2 *α*-pinene is really low (3.01 %) (Table 4). Furthermore, for the other analyzed samples, *α*-pinene was found in significantly lower concentrations (0.59 %–17.51 %, Table 4). The samples from Slavyanka (T2) also contained high amounts of *β*-pinene (Table 4). *α*-Phellandrene was detected only in one tree from Pirin (T5), with concentrations in different plant parts ranging as follows 49.61 % (MC) > 49.56 % (WT) > 43.37 (T) > 40.31 % (TT) (Table S1).

### 3.3 Total polyphenol, flavonoids content and radical scavenging activity of Pinus heldreichii

For the first time, the polyphenol, flavonoid content, and radical scavenging activity, of different parts of *P. heldreichii* (annual wood, biennial wood, leaf, M cones, and top of a twig) were investigated and the results are presented in Table 6. Many scientific studies have proven the beneficial effects of secondary metabolites from *Pinus* species, including terpenoids, volatile substances, flavonoids, polyphenols, ascorbic acid, and others. Extracts or EOs from the bark, needles, buds, and cones can be used for various diseases, such as rheumatism, inflammatory processes of the respiratory system, as an antitumor agent, antimicrobial, and antioxidant agent [24]. Despite the highlighted EO properties of *P. heldreichii*, the benefits of extracts of the whole parts (needles, twigs) are also significant. The content of valuable secondary metabolites with proven radical scavenging activity is shown in Table 6. There was a significant difference in the values of the investigated indicators depending on the sample type. The leaves had the highest values in terms of polyphenols (31.65 mg g^−1^) and flavonoids (11.20 mg g^−1^) (Table 6), determining the highest antiradical activity reported by the tests for the discoloration of DPPH and ABTS radicals expressed in % or Trolox equivalents. On the other hand, MC and TT have the lowest values for phenolic and flavonoid compounds, and accordingly, antiradical activity. The wood part is also characterized by high values for phenolic and flavonoid compounds, which determine the antiradical activity of their extracts. A slightly higher content of phenolic (22.72 mg g^−1^) and flavonoid (9.04 mg g^−1^) (Table 6) compounds was found in extracts from annual wood compared to extracts from biennial wood.

**Table 6:** Mean phenols (mg GAE g^−1^), flavonoids (mg QE g^−1^) DPPH inhibition (% DPPH inhibition for 30 min), DPPH TE (TE mg g^−1^), ABTS inhibition (% ABTS inhibition for 5 min), and ABTS TE (TE mg g^−1^).

Plant Part tissue	Phenols	Flavonoids	DPPH inhibition	DPPH TE	ABTS inhibition	ABTS TE
**Annual WT**	22.7 b	9.04 b	86.5 b	1.02 b	70.1 b	2.36 b
**Biennial WT**	18.6 c	7.24 c	86.5 b	1.02 b	62.8 c	2.07 c
**L**	31.6 a	11.20 a	91.3 a	1.08 a	87.9 a	3.06 a
**MC**	4.6 d	5.98 d	75.8 c	0.89 c	13.9 d	0.15 d
**TT**	4.5 d	7.02 c	85.4 b	1.01 b	16.9 d	0.27 d

*within each response variable, means followed by the same letter are not significantly different. MC - Male, cones; WT - wood of twigs; L – leaves (needles); TT – twigs tips. The letter groupings were done using Tukey's studentized range test method at 5 % level of significance.

Antiradical activity characterizes the ability of compounds to react with free radicals. Consequently, all test systems using a stable free radical (DPPH, ABTS, etc.) give information on the radical scavenging or antiradical activity. The pine leaves extract showed higher ABTS radical scavenging activity (77.03 % inhibition) and comparatively lower DPPH radical scavenging activity (26.59 % inhibition).

### 3.4 Antimicrobial activity of EOs of Pinus heldreichii

The antimicrobial activity of the tested *P. heldreichii* EOs is shown in Table 7. Overall, EOs extracted from different plant parts and populations of *P. heldreichii* exhibited different levels of activity. For example, the EO from T, T3 from Pirin Mountain showed good antimicrobial activity against *L. monocytogenes* and *B. cereus* (8.76 ± 0.58 mm and 6.33 ± 0.58 mm, respectively), while the EOs from T, T1 from Vitosha Mountain, WT, T6, T7, from Pirin Mountain, and MC, T1 showed good antimicrobial activity against *S. enterica* (8.76 ± 0.58 mm) and *P. aeruginosa* (6.33 ± 0.58 mm), respectively. Moreover, the EO of T, T6 of Pirin Mountain showed good activity against *E. coli* (10.33 ± 0.58 mm). Generally, the highest antimicrobial activity against *S. aureus* subsp. *aureus* was observed in EOs extracted from T from all locations (T1,T2, T4,T6), with inhibition zones of 5.67 ± 0.58 mm, respectively (Table 7). The best antimicrobial effect against *C. albicans* was observed in the EOs extracted from T, T1, T5, and MC, T1 (Table 7).

**Table 7:** Antimicrobial activity of EOs of *Pinus heldreichii*.

Samples, Tree №	Test organism**:** Average ± SD, inhibition halos diameters								
	*SA*^a^	*LM*	*BC*	*SE*	*PA*	*EC*	*CA*	*CG*	*CT*
MC, T1	5.33 ± 0.58	7.67 ± 0.58	5.33 ± 0.58	8.67 ± 0.58	6.33 ± 0.58	8.67 ± 0.58	6.33 ± 1.15	3.67 ± 0.57	3.33 ± 0.57
WT,T1	4.67 ± 0.58	7.67 ± 0.58	5.33 ± 0.58	7.67 ± 0.58	5.33 ± 0.58	8.67 ± 0.58	5.33 ± 0.58	3.67 ± 0.58	3.67 ± 0.58
WT,T4	4.67 ± 1.15	7.33 ± 0.58	4.67 ± 0.58	7.67 ± 1.16	5.33 ± 0.58	7.67 ± 0.58	5.33 ± 0.58	2.67 ± 0.58	2.33 ± 0.58
WT,T5	4.67 ± 0.58	8.33 ± 0.58	5.67 ± 0.57	8.67 ± 1.15	5.33 ± 0.58	7.67 ± 0.58	5.33 ± 0.58	2.67 ± 0.58	2.67 ± 0.58
T,T1	5.67 ± 0.58	8.33 ± 0.58	5.66 ± 0.58	8.67 ± 0.58	6.33 ± 0.58	8.67 ± 0.58	6.33 ± 1.15	3.67 ± 0.58	3.33 ± 0.58
T, T2	5.67 ± 0.58	7.67 ± 0.58	5.33 ± 0.58	7.67 ± 1.15	5.33 ± 0.58	7.67 ± 0.58	5.33 ± 0.58	2.67 ± 0.58	2.33 ± 0.58
T, T3	4.67 ± 0.58	5.33 ± 0.58	2.33 ± 0.58	7.67 ± 1.15	5.33 ± 0.58	7.67 ± 0.58	5.33 ± 0.58	2.67 ± 0.58	2.33 ± 0.58
T, T4	5.67 ± 0.58	6.33 ± 0.58	4.33 ± 0.58	7.67 ± 0.58	5.33 ± 0.58	8.67 ± 0.58	5.33 ± 0.58	3.67 ± 0.58	3.67 ± 0.58
T, T5	5.33 ± 0.57	8.33 ± 1.15	6.33 ± 0.58	7.67 ± 0.57	5.33 ± 0.58	8.67 ± 0.57	5.33 ± 0.57	3.67 ± 0.58	3.67 ± 0.58
T, T6	5.67 ± 0.58	7.67 ± 0.58	5.33 ± 0.58	8.33 ± 0.58	5.67 ± 0.58	8.33 ± 0.58	5.67 ± 0.58	3.33 ± 0.58	3.33 ± 0.58
T, T7	5.67 ± 0.58	8.76 ± 0.58	5.67 ± 0.58	8.33 ± 0.58	5.67 ± 0.58	10.33 ± 0.58	6.33 ± 0.58	3.67 ± 0.58	2.33 ± 0.58
**Antibiotic**									
Gentamycin	21.0 ± 1.00	24.33 ± 0.58	16.67 ± 0.57						
Cefoxitin				16.67 ± 1.53	17.0 ± 2.00	17.67 ± 1.53			
Fluconazole							18.53 ± 0.58	16.67 ± 1.53	17.33 ± 0.58

a *Staphylococcus aureus* subsp. *aureus* – SA; *Listeria monocytogenes* – LM; *Bacillus cereus* – BC; *Salmonella enterica* subsp*. enterica* – SE; *Pseudomonas aeruginosa* - PA; *Escherichia coli* – EC; *Candida albicans* – CA; *Candida glabrata* – CG; *Candida tropicalis* – CT. MC - Male, cones (microstrobiles); WT - wood of one-two-year-old twigs; T - whole twigs (leaves, wood, and twigs tip); TT – twigs tips.

## 4 Discussion

This study investigated of EOs composition and EOs yield in leaves (L), twigs (T), the twigs tips (TT), cones (M, F) (MC, FC), and wood of one-two-year old twigs (WT) of individual trees of *P. heldreichii* from Bulgarian populations. Furthemore, total polyphenol, flavonoids content and radical scavenging activity were discussed. Overall, previous analyses of *P. heldreichii* EO in Bulgaria exist, but this study stands out as the first comprehensive examination encompassing yield and EO composition of various plant parts of trees from this species.

### 4.1 EO yield of wood of one-two-year-old twigs (WT), leaves (L), non-grinded leaves (NL), the twigs tips (TT), cones (M, F)(MC, FC), and whole twigs (wood, leaves, the top of twigs) of *P. heldreichii*

The results of this study reveal differences in EO yield in different parts of *P. heldreichii* from one tree as well as between trees in the same population. The EO yield of different parts of *P. heldreichii* varied significantly from 0.09 % to 0.74 %. Varied results for EO yield were observed in literature data (Table 1). There are many reasons for the variation in results of EO yield as different period of collection of samples, different methods, genetic and physiological features, ecological conditions etc. For instance, dry leaf samples from Kosovo, EO yield varied from 0.2 to 0.3 %, while in twigs EO yield was 0.8–1.2 % (samples collected from July to September) [21]. Results close to those of Basholi-Salihu et al. [21] were found in fresh young shoots (1.25 %) and cones samples (0.85 %) collected in summer from Serbia [12]. The fresh leaf samples collected on May from Bulgaria the EO yield was 0.28 % [29], while for leaf samples from Calabria, Italy the yield was 0.2 % [27]. Our results of dry leaves EO yield shown high range from 0.09 % (T6; T7) to 0.39 % (T2) (Table 4). Furthermore, the similar ranges were found for other research plant parts (Table 4). In our previous study, for dry twigs samples collected in July, the yield was 0. 4 % [30] while in this study EO yield of T varied from 0.21 % (T7) to 0.6 % (T1). These examples demonstrate the presence of variations in EO yield of *P. heldreichii* which is not correlated with the period of sample collection and the geographical location of the populations. Overall, the results in this study are in partial agreement with previous reports on leaf EO yield [21,29] because results of EO yield were highly variable. However, most of the published data about *P. heldreichii* did not indicate the EO yield (Table 1), so we cannot compare our results. Furthermore, we did not find data on EO yield from different plant parts (T, WT, TT, MC, FC) in the same tree. In this sense, our study is the first of its kind.

As was mentioned in Section 2.2. to understand and demonstrate differences in EO yield due to grinding of samples, we included the two leaves samples (T3, T4) without grinding, only cut into pieces of 0.5–1 cm. According to most of the studies presented in Table 1 before extraction of the EOs, the samples were cut into small pieces (Table 1). However, previous research has shown that grinding of biomass from *Pinus* and *Junipers* has increased EO and reduced the distillation time [30,33,42]. Indeed, the above was supported from the results of this study; grinding leaves (T2, T3) did increase the EO yeld (0.25 %, 0.15 %) compared with non-grinded (0.12 %, 01 %) (Table 4). Furthermore, grinding in water (as done in this study) does not lead to any loss of EO as reported previously [30,33,42].

Overall, the EO yield in different parts of Bulgarian samples of *P. heldreichii* varied significantly. Apperantly, genetic and physiological factors mostly influence the EO yield because the samples were collected simultaneously, and they were extracted in the same way. Additionally, some samples from the same population have a high EO yield, while others have low EO yield. As known, *P. heldreichii* is distributed at high altitudes of about 1400–2200 masl and is characterized by high adaptability to extreme climates and tolerance to low temperatures [43]. A compensatory effect of *P. heldreichii* to extreme climatic conditions and low water supply is slowing its metabolism [44]. As a result of these compensatory reactions, the species produces different amounts of secondary metabolites.

### 4.2 Essential oils composition of wood of one-two-year-old twigs (WT), leaves (L), non-grinded leaves (NL), the twigs tips (TT), cones (M, F)(MC, FC), and whole twigs (wood, leaves, the top of twigs) of *P. heldreichii*

In this study L, WT, T, TT, MC, and FC of *P. heldreichii* were extracted and analyzed by GC-MS-FID. Overall, nine compounds of EOs were identified, and they represented 66.75–99.0 % of total EOs (Table 4; Table S1). Generally, limonene, α-pinene, *β*-caryophyllene, germacrene D, *β*-pinene, and *β*-myrcene were detected in all analyzed trees from the three populations (Table S1). We should note that these compounds (limonene, *β*-caryophyllene, and germacrene D) were not detected in all analyzed parts of trees. However, in some parts, they amount to 81.1 % of the total EO (Table 5). Overall, our result shows that components of EOs of different parts of trees varied, which are contrary to our working hypothesis. For example, between different parts in samples of trees distributed in Pirin Mountain limonene varied from 20.09 % (L, T6) to 66.3 % (MC, T6), *β*-caryophyllene from 1.06 % (MC T6) to 21.99 % (L, T5). Likewise, *α*-pinene ranged from 5.94 % (WT, T6) to 14.49 % T, T6), *β*-pinene from 0.0 % (L, T6) to 5.63 % T, T6, and germacrene D from 0.08 % (MC) to 40.80 % (L T6), respectively (Table 4, Table 5). As known, *P. heldreichii* in Pirin Mountain are distributed on marble limestone (calcareous) rocks, steep rocky terrain, on *Rendzinas* soils, and south or east exposure [10] (Table 2). The habitats are characterized by frequent and abundant rainfall, especially in winter [31].

Another example was the trees from Slavyanka Mountain where habitats of species are distributed on limestone and marble limestone, under the influence of the Continental-Mediterranean climate, by warm summers with a very low amount of rain [31].

The main compounds in different parts (Table 4, Table 5) range as follows: limonene from 1.9 % (L, T2) to 81.1 % (MC, T4), germacren D 0.0 % (MC, T4) to 39.40 % (NL, T3), *β*-caryophyllene 0.0 % (MC, T4) to 17.01 % (NL, T3), α-pinene 0.59 % (WT, T4) to 46.63 % (T, T2), and *β*-pinene 0.0 % (WT, T4) to 8.98 % (L, T2).

Similar variations were found in all studied samples and between different parts of trees. These examples demonstrate the high differences of EOs compositions in the parts of the species. It is important to point out that the EO profiles of *P. heldreichii* show a strong dependence on the individual characteristics of the tree and the studied part. There was no correlation between environmental factors, the geographical location, and the EO composition of *P. heldreichii*, because as shown in Table 2 the populations were under a Continental-Mediterranean climate and soils and basic rock were the same (Table 2). This inference agrees with the conclusion of Rajčević et al. [16] which indicates that the EO of *P. heldreichii* showed no correlation with bioclimatic parameters.

As a support of our conclusion, the % ratio of the main EO constituents of the grinded leaves of *P. heldreichii* can be grouped (Supll. Fig. 1). The lowest value for the nine constituents in the groups was 5 % of the total EO. Samples of leaves can be grouped into three chemotypes as follows:

**Chemotype (1)** - limonene (42.7/24.0/19.0/20.09); germacrene D (6.97/31.33/43.11/40.80); *β*-caryophyllene (21.99/10.85/17.01/29.98); *α*-pinene (8.71/10.91/7.97/6.02); for T5-T7, Pirin, and T1,Vitosha (Table 4, Table 5);

**Chemotype (2)** - *α*-pinene (44.61); germacrene D (29.41); *β*-pinene (8.98); for T2,Slavyanka;

**Chemotype (3)** - limonene (23.5/46.0); *α*-pinene (14.68/19.71); germacrene D (39.07/7.06); *β*-caryophyllene (11.85/13.48); *β*-pinene (5.88/6.22); for T3, T4 Slavyanka (Table 4, Table 5);

The same main composition of EO like our 3rd chemotype was found for samples of leaves from Greece [25,28] and Serbia [14] whose populations are geographically separated (allopatric). Limonene, *α*-pinene, germacrene D and *β*-caryophyllene were the most prevailing compounds in EO of other *Pinus* species such as *P. pinea* L., *P. radiata* D. Don, and *P. halepensis* Mill. [28,[45], [46], [47]], and *P. cembra* L [48].

However, differences were observed between grinded (L) and non-grinded leaves (NL) in the same samples per tree (Table 4, Table 5). EO yield of NL (T3, T4 Slavyanka) were very low compared to that in L. As a result of grinded or non grinded samples before the extraction, the compositions and the quantity of EOs of leaves were different (Table 4, Table 5). For example, *α*-pinene was 14.68 % (L) while for NL it was 4.15 %, *β*-pinene 5.88 % (L), and for NL it was 2.51 %, limonene was 23.5 % (L) and 16.3 % in NL, respectively (Table 4, Table 5). Apparently, post-harvest processes had resulted in a change in EO composition.

Comparison of the compositions of EOs of twigs is an important research interest (Table S1). Besides *α*-pinene, limonene, *β*-caryophyllene, and germacrene D, *α*-phellandrene was found in the EO of T. Because of its pleasing aromas, *α*-phellandrene is used as a fragrance compound [49]. *α*-Phellandrene was also found in TT, T5 (38.79 %), WT, T5 (43.68 %) and MC, T5 (49.61 %) (Table S1). Furthermore, it should be noted that very high level of the *α*-phellandrene is reported for the first time for *P. heldreichii* EO. According to Nikolić et al. [14] *α*-phellandrene was found in leaves samples from Serbia but its quantity was really low (0.00–0.09). The similar low quatity of *α*-phellandrene (0.4 %) was reported for *P. nigra* var. *calabrica* Schn. [27], while for *P.* *densiflora* Sieb. et Zucc. and *P. longaeva* Bailey their quatities are higher (13.2 % and 32.5 %, respectively) [50].

According to the results of this study the EOs composition of T of species would be classified as four chemotypes as follows.

• **Chemotype (1)** limonene (57.6/45.0/69.5/39.7); germacrene D (10.40/25.39/10.74/28.99); *α*-pinene (13.45/9.85/9.82/8.30); *β*-caryophyllene (6.53/7.43/7.22/11.77); for T1 Vitosha, T3 and T4 Slavyanka, T7 Pirin;
• **Chemotype (2)***α*-phellandrene (43.30); limonene (22.0); *α*-pinene (13.72); *β*-caryophyllene (5.79); for T5 Pirin;
• **Chemotype (3)***α*-pinene (46.63); germacrene D (22.91); *β*-pinene (7.26); for T2 Slavyanka;
• **Chemotype (4).** limonene (58.1); *α*-pinene (14.49); germacrene D (10.04); *β*-pinene (5.63); for T6, Pirin; (Table 4, Table 5; Table S1; Supp. Fig. 1).

The lowest value for the nine constituents in the above groups was 5 % of the total EO.

Previous studies on *P. heldreichii* essential oil has identified two chemical types in Bulgarian populations [26], two chemical types in samples from Montenegro and Serbia [11,14], and three chemical types in an another study from Serbia [16]. This indicates that there is a high degree of variability in the essential oil composition of this species. Our results are partly consistent with previous literature reports [11,14,26], which have also shown that there is a significant variation in EO compositions across different parts of the trees, as demonstrated in Table 4, Table 5, and Table S1.

However, the cited authors have only analyzed one sample per study or per tree, or combined the results and presented the result as average value, which does not give an accurate idea of the EOs differences in individual trees and is insufficient for drawing reliable conclusions. Results of this study show that genetic features, individual physiological characteristics of trees, and the method of extracting the essential oils are the main factors influencing EO composition. *Pinus heldreichii* is a survivor plant from the Tertiary and is now mainly restricted to certain areas (refugia) in the Bulgarian flora, including the Pirin Mountain and Slavyanka Mountains, where populations contain very old trees (over 500–600 years old) [10,[51], [52], [53]]. The two natural populations of *P. heldreichii* in Bulgaria (Pirin Mountain and Slavyanka Mountain) are situated near each other, making genetic drift possible. This indicates that much greater genetic diversity exists within the species in populations. This is not accidental because *P. heldreichii* survived in late glacial refugia, and genetic processes have continued [54,55].

The metabolism of *P. heldreichii* is affected by various factors, including ongoing genetic processes and individual physiological characteristics. Differences in EO yield and EO composition between individual trees from the three populations (Pirin Mountain, Slavyanka Mountain, and Vitosha Mountain) support this conclusion. The nuclear DNA that controls growth and the synthesis of proteins, enzymes, and functions differs among trees of the same species [44]. Additionally, differences in the synthesis of proteins have been found between individual trees and between populations [44]. The variations in EO composition and quantity can be attributed to the response of *P. heldreichii* to extreme climate conditions and individual genetic characteristics.

### 4.3 Total polyphenol and flavonoids content and radical scavenging activity of P. heldreichii

According to our research, needle extracts are distinguished with the highest anti-radical activity. This is most likely due to their reported highest content of total phenols and flavonoids. In a study of the antioxidant potential of needles of various representatives of the genus *Pinus*. Kurti et al. [56] found that the essential oil of *P. sylvestris* L. from Kosovo showed weak to moderate DPPH radical quenching potential, and the leaves' oils of *P. nigra* Arnold*, P. peuce* Griseb. and *P. heldreichii* showed weak activity against the same radical. In a similar study, the needle oil of *P. heldreichii* var. *leucodermis* from central Herzegovina showed weak DPPH radical activity [9]. There are few studies on the antiradical activity of alcoholic or other extracts of conifer leaves tissue. Koutsaviti et al. [23] investigated the antiradical activity of 46 species of the genus *Pinus*. Their study compared the essential oil obtained from needles with alcohol or organic extracts of leaves. They found a relatively high antioxidant activity of the essential oil and organic or alcoholic extracts of *P. heldreichii* compared to other genus *Pinus* subsec. *pinaster* representatives. This is one of the few published studies that revealed the potential of crude extracts of leaves, which confirms the need for more and deeper research.

### 4.4 Antimicrobial activity of EO of P. heldreichii

Overall, the tested EOs from different plant parts of *P. heldreichii* exhibited different levels of activity but EO from T (twigs) (all tested samples) had the highest antimicrobial activity against *S. aureus* subsp. *aureus.* This result was in agreement with that of a previous research on *P. heldreichii* EO [29]. The composition of tested EOs in this study were very different (Table 4, Table 5). The major EO constituents, *α*-pinene (46.3 %, T2), limonene (57.6 % T1; 69.5 % T4; 58.1 % T6), *α*-phellandrene (43.3 % T5), germacrene D and *β*-caryophyllene prevailed in varying amounts in T EO. *α*-Pinene, *β*-pinene and limonene have been reported to be responsible for the antimicrobial activity of *Pinus* EOs [6,29,56,57]. The exact interaction mechanism between *P. heldreichii* EO constituents and pathogens is not clear. Because we did not test individual EO constituents as pure substances, we cannot verify the exact contribution of each EO constituent to the observed antimicrobial effects. Furthermore, the antibacterial properties of EO cannot be explained by a single mechanism, and a variety of mechanisms have been proposed to explain how an EO affects bacterial cells [58]. The most significant mechanism of action of EOs against bacteria has been found to be the impairment of the structural and functional properties of the bacterial cell membrane. The ability of EOs to interact with the cell membrane and be absorbed by the cell is a result of their hydrophobic nature. After entering the bacterial cell, some essential oil components bind to hydrophobic protein sites and encourage modifications to the membrane structure. These modifications alter the fluidity and permeability of the cell membrane, cause the loss of essential intracellular components, obstruct nutrient absorption, and eventually cause cell lysis [59]. One of the main mechanisms under investigation is how lipophilic oil components alter the permeability of cell membranes of microorganisms. The cytoplasmic membrane is composed of a phospholipid bilayer, and damage to their integrity causes changes in the way the electron transport chain works, how nutrients are absorbed, how proteins and nucleic acids are produced, how cell contents coagulate, and how key enzymes for the microbial cell's energy metabolism are inhibited [[60], [61], [62]]. Due to changes in the composition of their cell walls, Gram-negative bacteria often exhibit greater resistance to the antimicrobial effects of EOs than Gram-positive bacteria [63]. For instance, researchers found that the leaves of various *Pinus* species exhibited moderate antimicrobial activity against both Gram-positive and Gram-negative bacteria, as well as fungi. Among these, the leaves of specific species such as *P. rigida* P. Mill., *P. caribaea* Morelet, *P. densiflora* Siebold & Zucc., and *P. thunbergii* Parl. were noted to have the most potent antimicrobial effects, particularly against the pathogens *Salmonella enteritidis* and *P. aeruginosa* [64]. In our previous study [30], we found that the EOs of *P. peuce* Griseb., *P. heldreichii*, and *P. mugo* Turra had antibacterial properties. *Pinus heldreichii* EOs were particularly active against Gram-negative bacteria such as *S. enterica* subsp. *enterica* and *E. coli*, with effects ranging from 3 to 8 mm. Similar variation in antimicrobial effects were observed in other *Pinus* EOs against a range of pathogens, including various strains of Gram-negative (*E. coli*) and Gram-positive (*E. faecalis*) bacteria, as well as yeasts (*C. albicans*, *C. krusei*) [56,57]. Furthermore, Politeo et al. [65] reported no antibacterial activity against *E. coli* and a strong dose-dependent antimicrobial action of *P. nigra* spp. *dalmatica* leaf EO (Croatia) against *C. albicans*. Other investigations have reported varying degrees of effectiveness in *P. sylvestris* EO, with moderate-to-low activity against *P. brevicompactum, P. citrinum*, and *P. crustosum*, and minimal to no *in vitro* antibacterial properties against bacteria *E. faecalis* and *E. coli*, and the yeast *C. albicans* [[66], [67], [68]].

## 5 Conclusions

This study represents the first comprehensive investigation of the various parts of the endemic plant *P. heldreichii*, and reveals significant variations in the EO profiles of different plant parts. Notably, this study has identified four chemotypes of EO for twigs and three chemotypes for leaves, which are likely attributable to genetic factors. Furthermore, the EO of twig tips (TT), male cones (MC), and wood of one-two-year-old twigs (WT) of the same trees were reported for the first time.

The total polyphenol, flavonoid content, and radical scavenging activity of tissues of annual twigs wood and biennial twigs wood, leaf tissue, MC tissue, and the twigs tips tissue is also reported for the first time in the accessible literature. The leaves and woods exhibited high values for phenolic and flavonoid compounds, which determine the antiradical activity of their extracts. These findings highlight the potential of *P. heldreichii* to provide EOs with varying compositions and bioactivities, making them suitable for nutraceutical, pharmacological, and potentially food additive applications.

Supplementary Materials (Table S1) “The EOs compounds (minimum (min) and maximum (max)) between plant parts of *Pinus heldreichii* from Bulgaria”, and Supplemental Fig. 1 (Graphical representation of the chemical types of *P. heldreichii* in this study).

## Funding

This research was funded by The Bulgarian National Science Fund (10.13039/501100003336BNSF), Project KP-06-H36/14 from December 17, 2019; and also by the Natural Products Utilization Research Unit, USDA-ARS, University, MS 38677, USA.

## Data Availability statement

Data on the compounds are available from the authors, and they will be made available on request.

### Institutional review board statement

Not applicable for studies not involving humans or animals.

## Informed consent statement

Not applicapble for studies not involving humans.

## CRediT authorship contribution statement

**Ivanka Semerdjieva:** Conceptualization, Data curation, Formal analysis, Funding acquisition, Investigation, Methodology, Project administration, Resources, Supervision, Writing – original draft. **Charles L. Cantrell:** Formal analysis, Investigation, Methodology, Resources, Software, Validation, Visualization, Writing – review & editing. **Valtcho D. Zheljazkov:** Conceptualization, Investigation, Methodology, Writing – original draft. **Tzenka Radoukova:** Investigation, Writing – review & editing. **Lyubka H. Koleva-Valkova:** Formal analysis, Investigation, Methodology, Resources, Software, Validation, Writing – review & editing. **Tess Astatkie:** Formal analysis, Investigation, Methodology, Resources, Software, Validation, Visualization, Writing – review & editing. **Miroslava Kačániová:** Formal analysis, Investigation, Methodology, Resources, Software, Validation, Writing – review & editing. **Daniela Borisova:** Investigation, Writing – review & editing.

## Declaration of competing interest

The authors declare that they have no known competing financial interests or personal relationships that could have appeared to influence the work reported in this paper.

## References

[1] Bader A., Flamini G., Cioni P.C., Morelli I. (2000). Composition of the essential oils from leaves, branches and cones of *Pinus laricio* Poiret collected in Sicily. Italy. J. Essent. Oil Res..

[2] Semiz G., Heijari J., Isik K., Holopainen J.K. (2007). Variation in needle terpenoids among *Pinus sylvestris* L. (Pinaceae) provenances from Turkey. Biochem. Syst. Ecol..

[3] Shi Z., Deng X., Zeng L., Shi S., Lei L., Xiao W. (2022). Acclimation strategy of Masson Pine (*Pinus massoniana*) by limiting flavonoid and terpenoid production under low light and drought. Int. J. Mol. Sci..

[4] Dziedziński M., Kobus-Cisowska J., Stachowiak B. (2021). *Pinus* species as prospective reserves of bioactive compounds with potential use in functional food - current state of knowledge. Plants.

[5] Judzentiene A., Kupcinskiene E. (2008). Chemical composition on essential oils from needles of *Pinus sylvestris* L. grown in Northern Lithuania. J. Essent. Oil Res..

[6] Mitić Z.S., Jovanović B., Jovanović S.Č., Mihajilov-Krstev T., Stojanović-Radić Z.Z., Cvetković V.J., Mitrović T.Lj, Marin P.D., Zlatković B.K., Stojanović G.S. (2018). Comparative study of the essential oils of four *Pinus* species: chemical composition, antimicrobial and insect larvicidal activity. Ind. Crops Prod..

[7] Bourgaud F., Gravot A., Milesi S., Gontier E. (2001). Production of plant secondary metabolites: a historical perspective. Plant Sci..

[8] (2016). The IUCN red list of threatened species. https://www.iucnredlist.org/species/42368/95725658.

[9] Maric S., Jukic M., Katalinic V., Milos M. (2007). Comparison of chemical composition and free radical scavenging ability of glycosidically bound and free volatiles from Bosnian Pine (*Pinus heldreichii* Christ. var. *leucodermis*). Molecules.

[10] Valchev V., Rusakova V., Biserkov V. (2015). Red Book in Bulgaria.

[11] Bojović S., Nikolić B., Ristić M., Orlović S., Veselinović M., Rakonjac L., Dražić D. (2011). Variability in chemical composition and abundance of the rare Tertiary relict *Pinus heldreichii* in Serbia. Chem. Biodivers..

[12] Menković N.R., Ristić M.S., Samardžic′ Z.J., Kovačević N.N., Tasić S.R. (1993). Investigations of relict *Pinus* species. II. The Essential oil of *Pinus heldreichii*. Acta Hort.

[13] Simić N., Palić R., Andelković S., Vajs V., Milosavljević S. (1996). Essential oil of *Pinus heldreichii*. needles. J. Essent. Oil Res..

[14] Nikolić B., Ristić M., Bojović S., Marin P.D. (2007). Variability of the needle essential oils of *Pinus heldreichii* from different populations in Montenegro and Serbia. Chem. Biodivers..

[15] Nikolić B., Ristić M., Bojović S., Krivošej Z., Matevski V., Marin P.D. (2015). Population variability of essential oils of *Pinus heldreichii* from the Scardo-Pindic mountains Ošljak and Galičica. Chem. Biodivers..

[16] Rajčević N., Nikolić B., Marin P.D. (2019). Different responses to environmental factors in terpene composition of *Pinus heldreichii* and *P. peuce*: ecological and chemotaxonomic considerations. Arch. Biol. Sci..

[17] Apetrei C.L., Tuchilus C., Aprotosoaie A.C., Oprea A., Malterud K.E., Miron A. (2011). Chemical, antioxidant and antimicrobial investigations of *Pinus cembra* L. bark and needles. Molecules.

[18] Apetrei C.L., Spac A., Brebu M., Tuchilus C., Miron A. (2013). Composition, and antioxidant and antimicrobial activities of the essential oils of a full-grown *Pinus cembra* L. tree from the Calimani Mountains (Romania). J. Serb. Chem. Soc..

[19] Sacchetti G., Maietti S., Muzzoli M., Scaglianti M., Manfredini S., Radice M., Bruni R. (2005). Comparative evaluation of 11 essential oils of different origin as functional antioxidants, antiradicals and antimicrobials in foods. Food Chem..

[20] Graikou K., Gortzi O., Mantanis G., Chinou I. (2012). Chemical composition and biological activity of the essential oil from the wood of *Pinus heldreichii* Christ. var. *leucodermis*. Eur. J. Wood Wood Prod..

[21] Basholli-Salihu M., Schuster R., Hajdari A., Mulla D., Viernstein H., Behxhet Mustafa B., Mueller M. (2017). Phytochemical composition, anti-inflammatory activity and cytotoxic effects of essential oils from three *Pinus* spp. Pharm. Biol..

[22] Yener H.O., Saygideger S.D., Sarikurkcu C., Yumrutas O. (2014). Evaluation of antioxidant activities of essential oils and methanol extracts of *Pinus* species. J. Essent. Oil Bear. Plants.

[23] A. Koutsaviti, S. Toutoungy, R. Saliba, S. Loupassaki, O. Tzakou, V. Roussis, E. Ioannou, Antioxidant potential of Pine Needles: a systematic study on the essential oils and extracts of 46 species of the Genus *Pinus*. Foods. 10, 142. 10.3390/foods10010142. PMC7827367 33445574

[24] Popescu D.I., Lengyel E., Apostolescu F.G., Soare L.C., Botoran O.R., Șuțan N.A. (2022). Volatile compounds and antioxidant and antifungal activity of bud and needle extracts from three populations of *Pinus mugo* Turra growing in Romania. Horticulturae.

[25] Petrakis P.V., Tsitsimpikou C., Tzakou O., Couladis M., Vagias C., Roussis V. (2001). Needle volatiles from five *Pinus* species growing in Greece. Flavour Fragrance J..

[26] Naydenov K., Tremblay F., Bergeron Y., Alexandrov A., Fenton N. (2005). Dissimilar patterns of *Pinus heldreichii* Christ. populations in Bulgaria revealed by chloroplast microsatellites and terpenes analysis. Biochem. Syst. Ecol..

[27] Bonesi M., Menichini F., Tundis R., Loizzo M.R., Conforti F., Passalacqua N.G., Statti G.A., Menichini F. (2010). Acetylcholinesterase and butyrylcholinesterase inhibitory activity of *Pinus* species essential oils and their constituents. J. Enzyme Inhib. Med. Chem..

[28] E. Ioannou, A. Koutsaviti, O. Tzakou, V. Roussis, The genus *Pinus*: a comparative study on the needle essential oil composition of 46 pine species. Phytochem. Rev. 13, 741–768. 10.1007/s11101-014-9338-4.

[29] Mitić Z.S., Jovanović B., Jovanović S.Č., Stojanović-Radić Z.Z., Mihajilov-Krstev T., Jovanović N.M., Nikolić B.M., Marin P.D., Zlatković B.K., Stojanović G.S. (2019). Essential oils of *Pinus halepensis* and *P. heldreichii*: chemical composition, antimicrobial and insect larvicidal activity. Ind. Crops Prod..

[30] Semerdjieva I.B., Radoukova T., Cantrell C.L., Astatkie T., Kacaniova M., Borisova D., Zheljazkov V.D. (2022). Essential oil composition of *Pinus heldreichii* Christ., *P. peuce* Griseb., and *P. mugo* Turra as a function of hydrodistillation time and evaluation of its antimicrobial activity. Ind. Crops Prod..

[31] Velev St, Kopralev I. (2002). Geography of Bulgaria: Physical Geography and Socio-Economic Geography.

[32] Thiers B. (2012). http://sweetgum.nybg.org/ih/.

[33] Zheljazkov V.D., Astatkie T., Schlegel V. (2012). Effects of distillation time on the *Pinus ponderosa* essential oil yield, composition, and antioxidant activity. Hortscience.

[34] Adams R. (2009).

[35] Singleton V.L., Rossi J.A. (1965). Colorimetry of total phenolics with phosphomolybdic phosphotungstic acid reagents. Am. J. Enol. Vitic..

[36] Koleva-Valkova L., Piperkova N., Petrov V., Vassilev A. (2017). Biochemical responses of Peach leaves infected with *Taphrina deformans* Berk./Tul. Acta Univ. Agric. Silvic. Mendel. Brun..

[37] Zhishen J., Mengcheng T., Jianming W. (1999). The determination of flavonoid contents in mulberry and their scavenging effects on superoxide radicals. Food Chem..

[38] Brand-Williams W., Cuvelier M.E., Berset C. (1995). Use of free radical method to evaluate antioxidant activity. LWT – Food Sci. Technol..

[39] Xiao F., Xu T., Lu B., Liu B. (2020). Guidelines for antioxidant assays for food components. Food Frontiers.

[40] SAS Institute Inc (2014).

[41] Montgomery D.C. (2020).

[42] Semerdjieva I., Shiwakoti I., Cantrell Ch, Zheljazkov V., Astatkie T., Schlegel V., Radoukova T. (2019). Hydrodistillation extraction kinetics regression models for essential oil yield and composition in *Juniperus virginiana*. J. excelsa, and J. sabina. Molecules..

[43] Lazarević J., Menkis A. (2020). Fungal diversity in the phyllosphere of *Pinus heldreichii* H. Christ - an endemic and high-altitude pine of the Mediterranean region. Diversity.

[44] Anev S., Panayotov M., Tsvetanov N., Gogushev G., Tsavkov E., Zlatanov T., Anev S., Ivanova A., Nedelin T., Zafirov N., Aleksandrov N., Dountchev A., Vasileva P., Shishkova V., Stoyanov B., Sotirova N., Vatov A., Bebi P., Yurukov S. (2016). Mountain Coniferous Forests in Bulgaria – Structure and Natural Dynamics.

[45] Amri I., Hamrouni L., Hanana M., Gargouri S., Fezzani T., Jamoussi B.B. (2013). Chemical composition, physico-chemical properties, antifungal and herbicidal activities of *Pinus halepensis* Miller essential oils. Biol. Agric. Hortic..

[46] Amri I., Kouki H., Mabrouk Y., Hanana M., Jamoussi B., Hamrouni L. (2021). Essential oils of Tunisian *Pinus radiata* D. Don, chemical composition and study of their herbicidal activity. Vietnam J. Chem..

[47] Amri I., Khammassi M., Gargouri S., Hanana M., Jamoussi B., Hamrouni L., Mabrouk Y. (2022). Tunisian Pine essential oils: chemical composition, herbicidal and antifungal properties. J. Essent. Oils Bear. Plants.

[48] Lis A., Kalinowska A., Krajewska A., Mellor K. (2016). Chemical composition of the essential oils from different morphological parts of *Pinus cembra* L. Chem. Biodivers..

[49] G. İşcan, N. Kirimer, F. Demirchi, F. Demirchi, Y. Noma, H. Can Başer, Biotransformation of (−)-(R)-α-phellandrene: Antimicrobial activity of its major metabolite. Chem. Biodivers. 9(8), 1525-1532. 10.1002/cbdv.201100283. 22899613

[50] Tsitsimpikou Ch, Petrakis P.V., Ortiz A., Harvala C., Roussis V. (2001). Volatile needle terpenoids of six *Pinus* species. J. Essent. Oil Res..

[51] Panayotov M., Bebi P., Trouet V., Yurukov S. (2010). Climate signal in tree-ring chronologies of *Pinus peuce* and *Pinus heldreichii* from the Pirin Mountains in Bulgaria. Trees (Berl.).

[52] Rangelova P., Panayotov M. (2013). Structure of old-growth *Pinus heldreichii* forests in Pirin mountains. Bulg. J. Agric. Sci..

[53] Vasileva P., Panayotov M. (2016). Dating fire events in *Pinus heldreichii* forests by analysis of tree ring cores. Dendrochronologia.

[54] Levanič T., Jevšenak J., Hafner P. (2020). Stable isotopes reveal climate signal hidden in tree rings of endemic Balkan Pines. Atmosphere.

[55] Willis K. (1994). The vegetational history of the Balkans. Quat. Sci. Rev..

[56] F. Kurti, A. Giorgi, G. Beretta, B. Mustafa, F. Gelmini, C. Testa, S. Angioletti, L. Giupponi, E. Zilio, D. Pentimalli, A. Hajdari, Chemical composition, antioxidant and antimicrobial activities of essential oils of different *Pinus* species from Kosovo. J. Essent. Oil Res. 31(4), 263–275. 10.1080/10412905.2019.1584591.

[57] M. Karapandzova, G. Stefkov, I. Cvetkovikj, E. Trajkovska-Dokik, A. Kaftandzieva, S. Kulevanova, Chemical composition and antimicrobial activity of the essential oils of *Pinus peuce* (Pinaceae) growing wild in R. Macedonia. Nat. Prod. Commun. 9(11), 1934578X1400901. 10.1177/1934578x1400901124. 25532297

[58] Nazzaro F., Fratianni F., De Martino L., Coppola R., De Feo V. (2013). Effect of essential oils on pathogenic bacteria. Pharmaceuticals.

[59] Drosinos E.H., Skandamis P.N., Mataragas M., Toldrá F. (2009). Safety of Meat and Processed Meat. Food Microbiol. Food Saf.

[60] Barbosa L.N., Alves F.C.B., Andrade B.F.M.T., Albano M., Rall V.L.M., Fernandes A.A.H., Buzalaf M.A.R., Leite A. de L., de Pontes L.G., dos Santos L.D., Fernandes Junior A. (2020). Proteomic analysis and antibacterial resistance mechanisms of *Salmonella Enteritidis* submitted to the inhibitory effect of *Origanum vulgare* essential oil, thymol and carvacrol. J. Proteomics.

[61] Burt S. (2004). Essential oils: their antibacterial properties and potential applications in foods—a review. Int. J. Food Microbiol..

[62] D.P. Ilić, L.P. Stanojević, D.Z. Troter, J.S. Stanojević, B.R. Danilović, V.D. Nikolić, L.B. Nikolić, Improvement of the yield and antimicrobial activity of fennel (*Foeniculum vulgare* Mill.) essential oil by fruit milling. Ind. Crops Prod. 142, 111854. 10.1016/j.indcrop.2019.111854.

[63] Behbahani B.A., Noshad M., Falah F. (2019). F. Cumin essential oil: phytochemical analysis, antimicrobial activity and investigation of its mechanism of action through scanning electron microscopy. Microb. Pathog..

[64] E.J. Hong, K.J. Na, I.G. Choi, K.C. Jeung, E.B. Jeung, Antibacterial and antifungal effects of essential oils from coniferous trees. Biol. Pharm. Bull. 27(6), 863–866. 10.1248/bpb.27.863. 15187434

[65] Politeo O., Skocibusic M., Maravic A., Ruscic M., Milos M. (2011). Chemical composition and antimicrobial activity of the essential oil of endemic Dalmatian Black Pine (*Pinus nigra* ssp. *dalmatica*). Chem. Biodivers..

[66] S. Felšöciová, M. Kačániová, E. Horská, N. Vukovič, L. Hleba, J. Petrová, K. Rovná, M. Stričík, Z. Hajduová, Antifungal activity of essential oils against selected terverticillate penicillia. Ann. Agric. Environ. Med. 22(1), 38–42. 10.5604/12321966.1141367. 25780826

[67] K.A. Hammer, C.F. Carson, T.V. Riley, Antimicrobial activity of essential oils and other plant extracts. J. Appl. Microbiol. 86(6), 985–990. 10.1046/j.1365-2672.1999.00780.x. 10438227

[68] Tullio V., Nostro A., Mandras N., Dugo P., Banche G., Cannatelli M.A., Cuffini A.M., Alonzo V., Carlone N.A. (2007). Antifungal activity of essential oils against filamentous fungi determined by broth microdilution and vapour contact methods. J. Appl. Microbiol..

