# Supplementary figures and images for: Chemical profile, antioxidant and antimicrobial activity of Pinus heldreichii Christ. Distributed in Bulgaria
Source: Heliyon. 2023 Dec 10;10(1):e22967. doi: 10.1016/j.heliyon.2023.e22967 (PMC10770424; doi:10.1016/j.heliyon.2023.e22967)

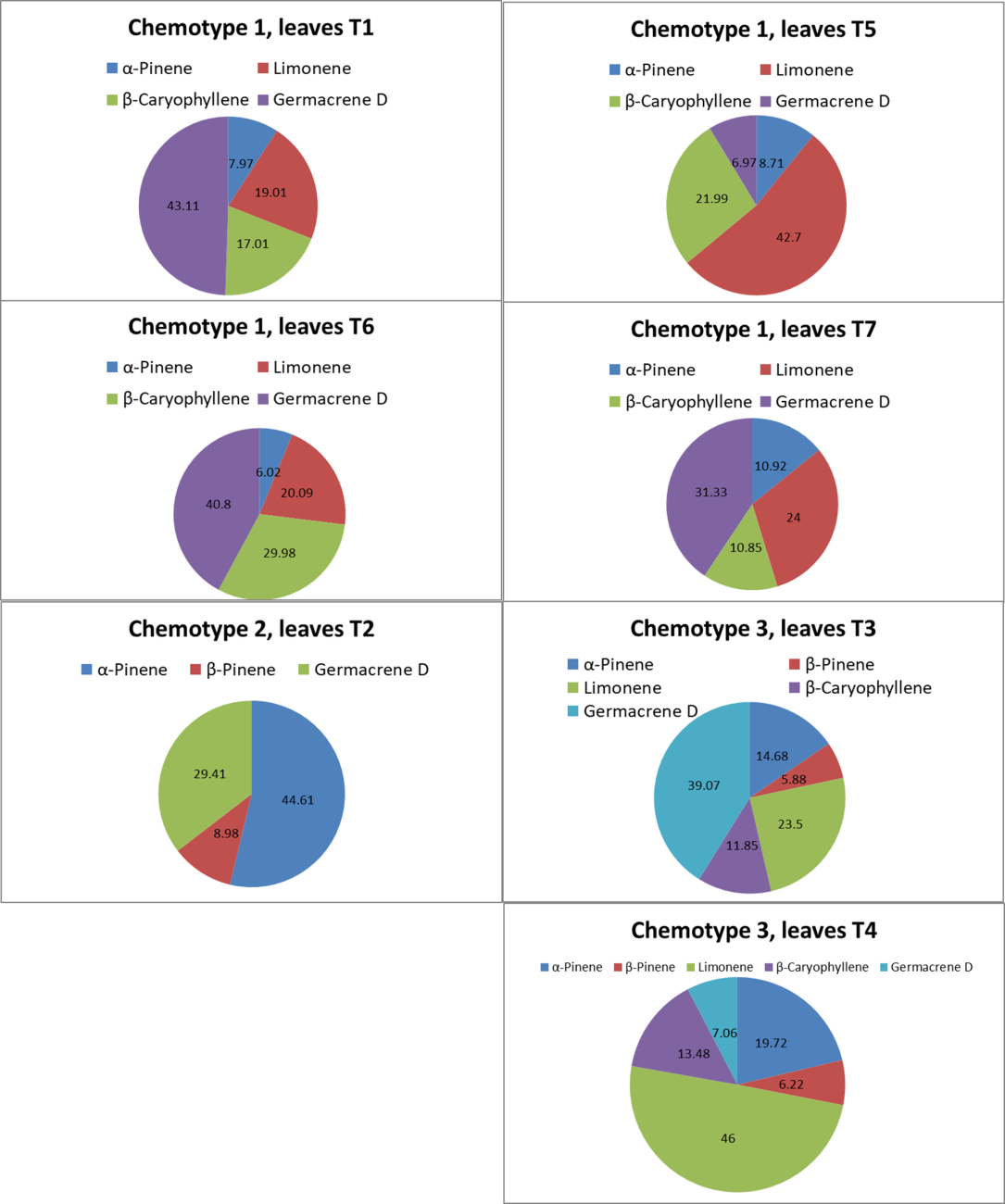


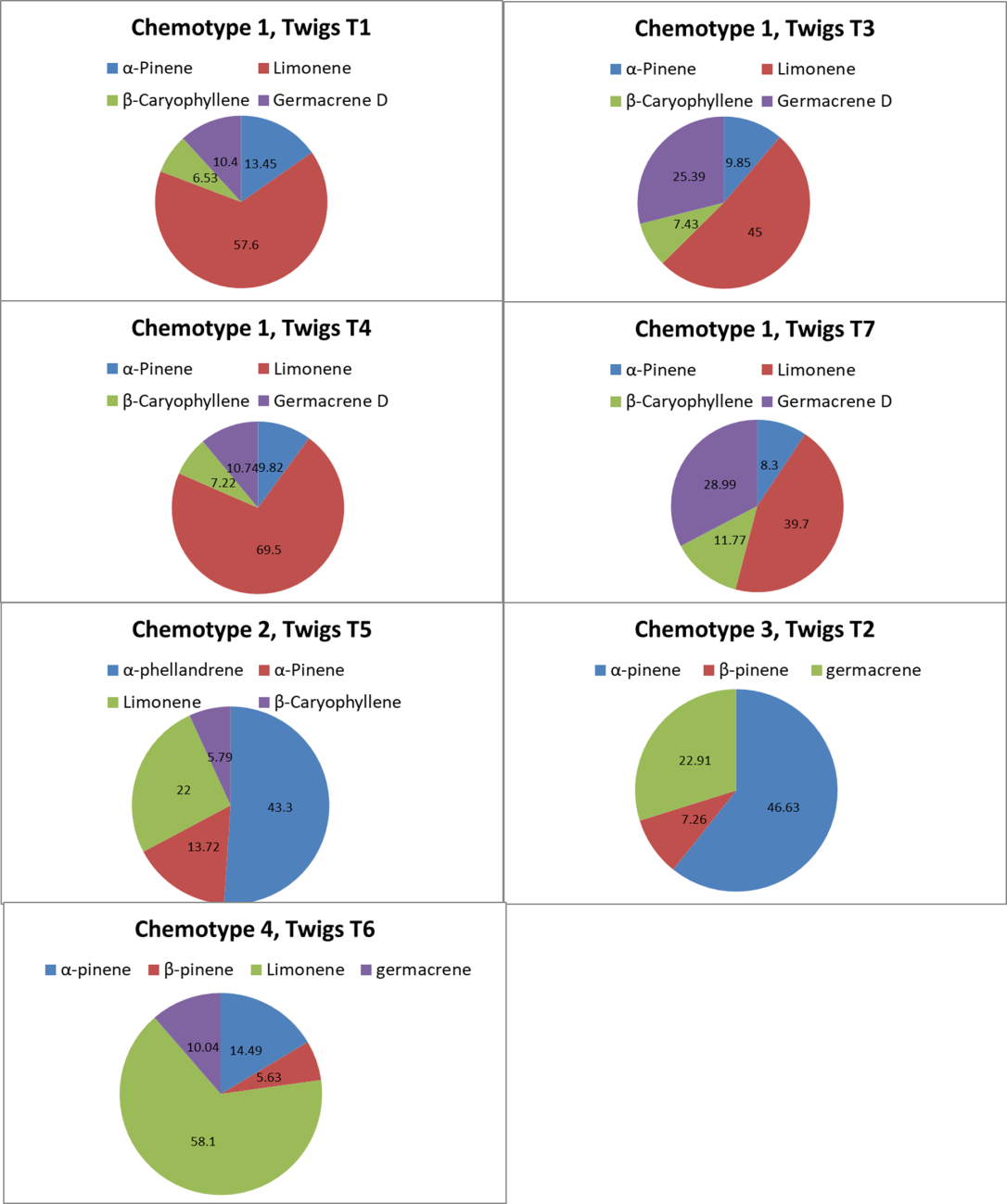

Supplement: Supplementary file 1 [file mmc1.docx]
