# Supplementary material for: Chemical profile, antioxidant and antimicrobial activity of Pinus heldreichii Christ. Distributed in Bulgaria
Source: Heliyon. 2023 Dec 10;10(1):e22967. doi: 10.1016/j.heliyon.2023.e22967 (PMC10770424; doi:10.1016/j.heliyon.2023.e22967)
Supplement: Supplementary file 2 [file mmc2.docx]

Table S1. The EOs compounds (minimum (min) and maximum (max)) between plant parts of *Pinus heldreichii* from Bulgaria.

| Population, № tree | Plant part | *α*-pinene | camphene | *β-*pinene | *β-*myrcene | *α*-phellandrene | limonene | bornyl acetate | *β*-caryophyllene | germacrene D |
| --- | --- | --- | --- | --- | --- | --- | --- | --- | --- | --- |
| Vitosha, T1 | WT | 7.88-10.07 | nd | 0.58-0.62 | 2.08-2.14 | nd | 78.76-79.37 | nd | 3.83-3.81 | 1.42-1.51 |
|  | T | 12.78-14.14 | nd | 2.49-2.81 | 1.84-2.1 | nd | 52.2-63.07 | 0.51-0.7 | 5.99-7.1 | 6.69-15.35 |
|  | L | 4.52-12.39 | nd | 1.81-4.04 | 0.41-1.22 | nd | 13.69-24.22 | nd | 15.44-20.48 | 36.57-50.42 |
|  | MC | 17.44-17.59 | nd | 0.87-0.9 | 1.7-1.81 | nd | 67.96-71.63 | 0.56-0.67 | 2.75-3.01 | 1.12-1.48 |
| Slavyanka. T2-T4 | T | 7.93-54.47 | nd | 2.20-8.34 | 0.94-2.45 | nd | 3.49-74.79 | 0.0-0.82 | 3.7-7.82 | 3.75-28.23 |
|  | L | 1.87 -45.88 | nd | 0.0-9.22 | 0.0-2.82 | nd | 1.87-48.05 | 0.0-0.34 | 4.22-27.9 | 5.46-40.01 |
|  | TT | 6.01-39.1 | nd | 5.786 | 0.76-1.83 | nd | 25.52-48.12 | 2.46-3.76 | 3.05-7.38 | 9.63-23.95 |
|  | WT | 0.49-3.5 | nd | 0.0-0.37 | 0.0-0.98 | nd | 19.32-49.38 | 0.0-1.69 | 0.0-7.1 | 0.0-10.91 |
|  | MC | 9.83-29.42 | 0.0-0.45 | 0.0-1.56 | 0.0-1.66 | nd | 40.31-83.09 | 0.0-2.34 | 0.0-2.04 | 0.0-1.33 |
|  | NL | 3.78-4.54 | nd | 2.52 | nd | nd | 16.09-16.49 | nd | 17.01-17.02 | 38.98-39.82 |
| Pirin, T5-T7 | TT | 6.29-9.92 | 0.0-0.50 | 0.0-3.04 | 0.0-2.26 | 0.0-38.79 | 21.07-57.3 | 1.6-5.25 | 5.68-6.47 | 0.08-8.17 |
|  | T | 6.65-17.41 | nd | 2.02-6.09 | 1.06-2.07 | 0.0-43.48 | 21.15-58.88 | nd | 3.31-17.34 | 2.40-38.08 |
|  | WT | 4.73-11.40 | 0.0-0.42 | 0.79-3.48 | 0.0-2.21 | 0.0-50.33 | 22.24-65.11 | 0.0-2.12 | 0.99-7.52 | 0.75-4.84 |
|  | L | 5.50-11.71 | 0.0-0.77 | 0.0-4.28 | 0.0-1.82 | nd | 20.74-48.05 | nd | 10.49-30.01 | 5.46-42.77 |
|  | MC | 7.47-18.48 | 0.0-0.923 | 1.93-4.33 | 0.99-1.78 | 0.0-49.61 | 31.38-67.45 | 0.0-2.93 | 0.0-9.7 | 0.0-7.53 |
|  | FC | 3.83-11.04 | nd | 0.35-0.82 | 0.99-2.17 | nd | 45.23-62.09 | 0.73-1.42 | 3.58-6.11 | 5.6-8.61 |

nd – no detected; MC - Male, cones; FC – Female, cones; WT - wood of one-two year-old twigs; T - whole twigs (leaves, wood, and twigs tip); L – leaves (needles) grinded; NL - non-grinded leaves.
